# LRP6 targeting suppresses gastric tumorigenesis via P14^ARF^–Mdm2–P53-dependent cellular senescence

**DOI:** 10.18632/oncotarget.22876

**Published:** 2017-12-04

**Authors:** Haibin Wang, Guoxing Xu, Zhengjie Huang, Weizheng Li, Huali Cai, Yunda Zhang, Disheng Xiong, Gang Liu, Shengjie Wang, Zengfu Xue, Qi Luo

**Affiliations:** ^1^ Department of Gastrointestinal Surgery, Xiamen Cancer Hospital, The First Affiliated Hospital of Xiamen University, Xiamen 361003, Fujian, China; ^2^ Department of Endoscopy Center, The First Affiliated Hospital of Xiamen University, Xiamen 361003, Fujian, China; ^3^ Department of Gastrointestinal Surgery, First Clinical Medical College of Fujian Medical University, Fuzhou 350004, China; ^4^ Department of Cancer Prevention, Diagnosis and Treatment, Xiamen Cancer Hospital, The First Affiliated Hospital of Xiamen University, Xiamen 361003, Fujian, China

**Keywords:** NLRP6, gastric cancer, senescence, P14^ARF^, P53

## Abstract

NLRP6, a member of the Nod-like receptor family, protects against chemically induced intestinal injury and colitis-associated colon cancer. However, the cellular mechanisms involved in this NLRP6-mediated protection remain unclear. Here, we show that NLRP6 was down-regulated in approximately 75% of primary gastric cancer cases and exhibited significant associations with advanced clinical-stage lymph node metastasis and poor overall survival. Functional studies established that ectopic overexpression or down-regulation of NLRP6 inhibited cancer cell proliferation by inducing cell cycle arrest at the G1 phase via P21 and Cyclin D1 both *in vitro* and *in vivo*. Activation of the P14^ARF^-P53 pathway played a crucial role in the observed cellular senescence. We further demonstrated that ectopic overexpression of NLRP6 combined with inactivation of NF-κB(p65) and Mdm2 activates P14^ARF^-P53 to promote the senescence of gastric cancer cells. These findings indicate that NLRP6 functions as a negative regulator of gastric cancer and offer a potential new option for preventing gastric cancer.

## INTRODUCTION

Gastric cancer is one of the most common causes of cancer-related death [1]. The majority of gastric cancer patients are diagnosed at an advanced stage and typically exhibit extensive tumor invasion and distant lymph node metastasis [2, 3]. Although great efforts have been made to improve early diagnosis rates and provide advanced treatments for patients with gastric cancer, the prognosis of gastric cancer patients remains poor [4, 5]. Therefore, a better understanding of the molecular mechanisms of gastric cancer pathogenesis is essential for developing effective targeted treatments.

Inflammasomes are multiprotein complexes consisting of one of several upstream NOD-like receptor (NLR) proteins, the adaptor protein apoptosis-associated speck-like protein containing CARD (ASC) and the effector protein caspase-1 [6]. Upon receipt of specific transcriptional and post-translational signals, inflammasomes are assembled and activated by autocleavage of pro-caspase-1 and promote the catalytic activation of IL-1β and IL-18 [7]. The members of the NLR family, including NLRP6, are involved in recognizing microbes and/or tissue injury [8]. The NLRP6 inflammasome is a key regulator of colonic homeostasis [9–11], and NLRP6 is predominantly expressed in intestinal epithelial cells, including goblet cells [10–12], where it plays central roles in mucus self-renewal, cell proliferation, and mucus secretion [11, 13, 14]. Mice deficient in NLRP6, ASC, or caspase-1 exhibit a distinct form of dysbiosis characterized by intestinal autoinflammation, inflammation-induced colorectal cancer and features of metabolic syndrome [8, 10, 15–17]. It has been postulated that factors secreted by wild-type microbiota activate the NLRP6 inflammasome, but no such factors have been identified to date. Moreover, the mechanisms by which NLRP6 deficiency leads to intestinal inflammation and tumorigenesis are poorly understood.

The current report revealed that NLRP6 is down-regulated in gastric cancer cell lines and tumor tissue samples. Furthermore, low NLRP6 expression was associated with poor prognosis in gastric cancer. These findings were further validated mechanistically by *in vitro* and *in vivo* NLRP6 overexpression and knockdown.

## RESULTS

### NLRP6 is down-regulated in gastric cancer tissues and gastric cancer cell lines

**Figure 1:**
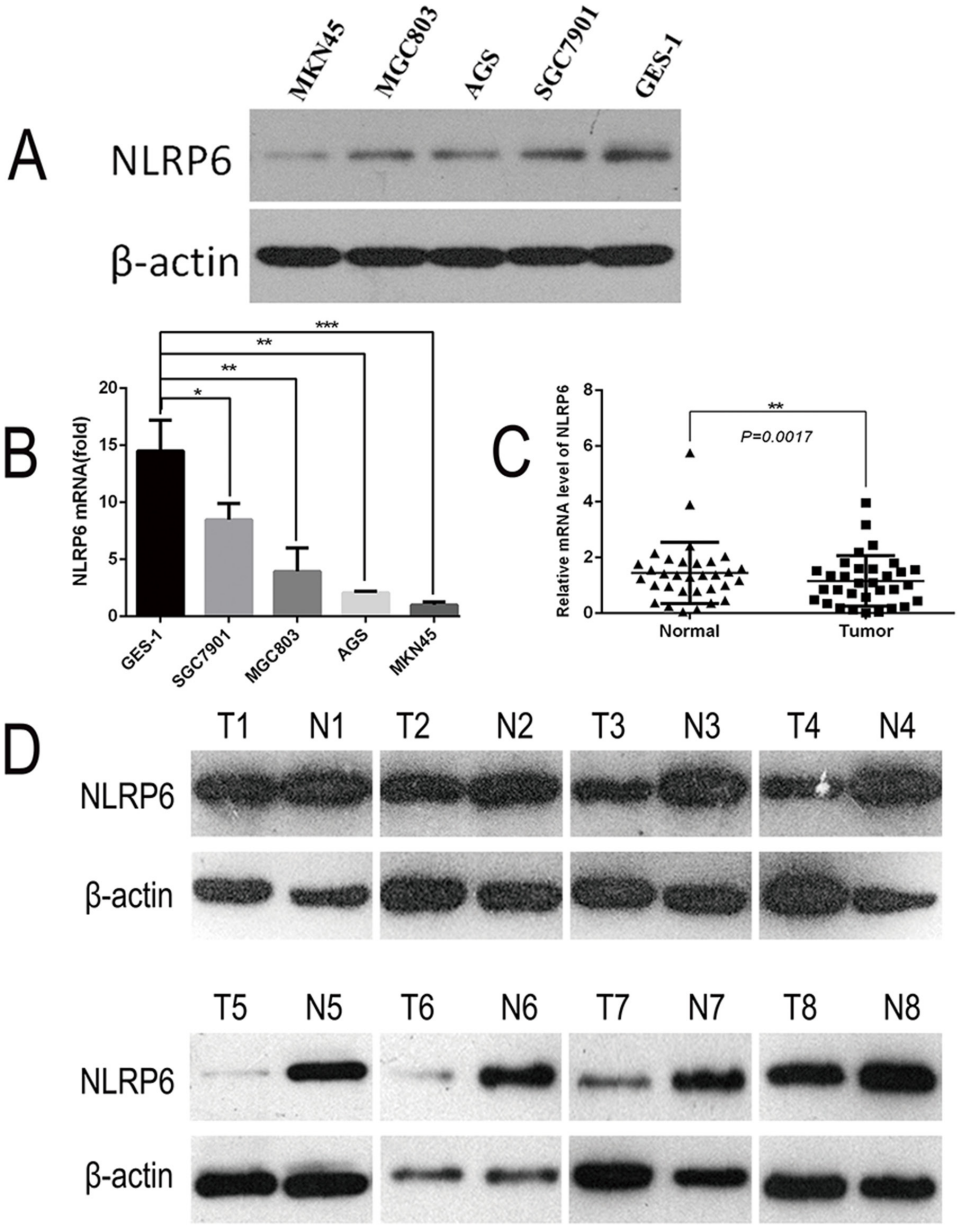
Expression of NLRP6 in gastric cancer cell lines **(A)** NLRP6 protein expression was examined in gastric cancer cell lines via Western blotting. **(B)** NLRP6 mRNA expression was examined in gastric cancer cell lines via RT-PCR. ^*^P<0.05, ^**^P<0.01, ^***^P<0.001. **(C)** NLRP6 mRNA expression was examined in gastric cancer tissues and their adjacent non-neoplastic tissues via RT-PCR. ^*^P<0.05. **(D)** NLRP6 protein expression was examined in gastric cancer tissues and their adjacent non-neoplastic tissues via Western blotting.

To examine the potential relationship between the expression of NLRP6 and the progression of gastric cancer, NLRP6 expression was investigated in the MKN45, SGC7901, MGC803 and AGS gastric cell lines. The results were compared to the normal gastric mucosa epithelium cell line GES-1. Expression was measured using both qPCR and Western blotting (Figure 1A, 1B). Additionally, NLRP6 mRNA expression was evaluated in gastric tumor tissues and in the surrounding nontumor tissues from 32 surgical specimens. The qPCR results revealed that NLRP6 was down-regulated in 24/32 (75%) of primary gastric tumors compared to their nontumor counterparts (*P*<0.05, Figure 1C). Western blot analysis confirmed down-regulation of NLRP6 protein in tumors (Figure 1D).

### NLRP6 expression is a key prognostic factor for patient survival

**Figure 2:**
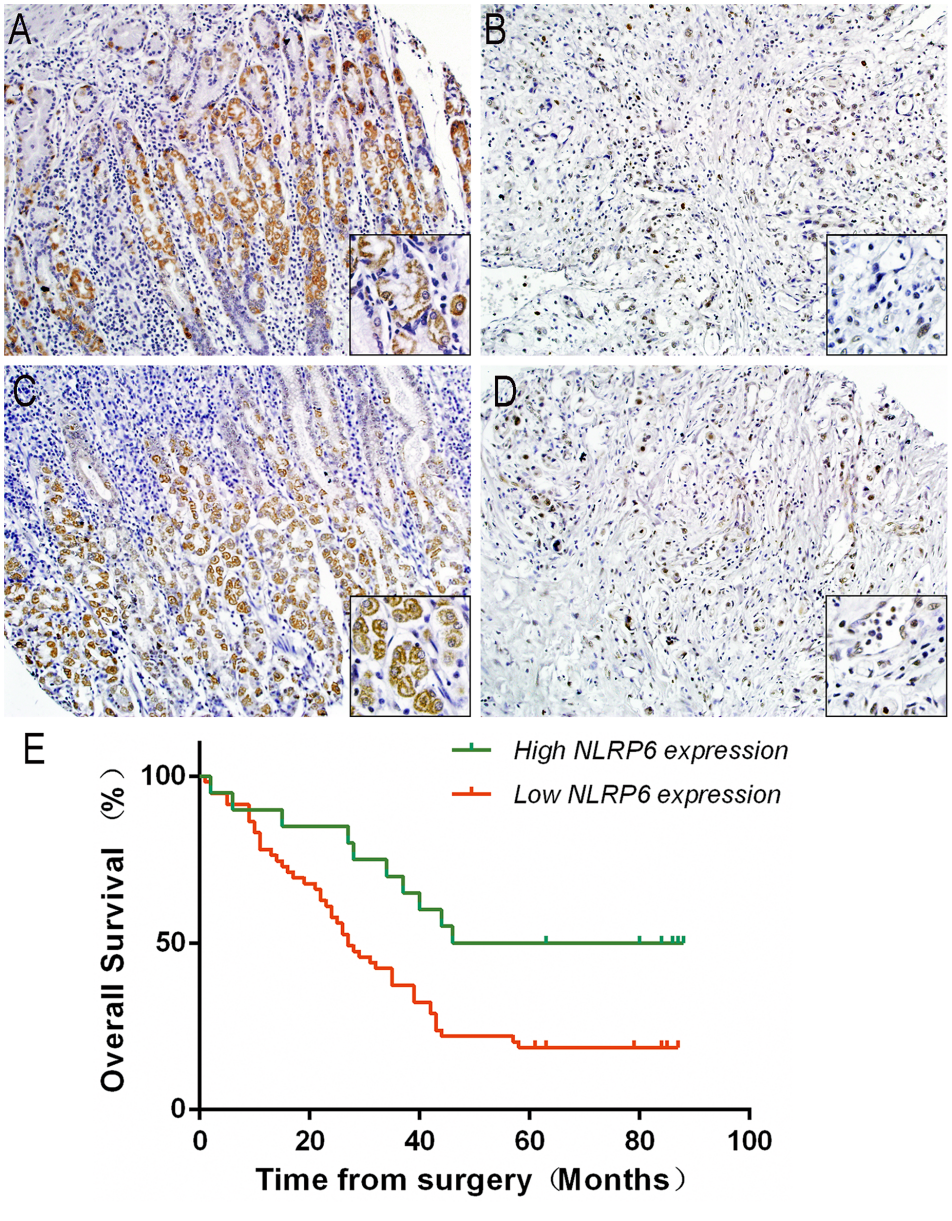
NLRP6 protein expression in gastric cancer surgical specimens evaluated by immunohistochemistry (×200, The lower right×400) and Kaplan-Meier survival curves of gastric cancer patients after gastrectomy **(A** and **B)** High NLRP6 expression was observed in adjacent non-neoplastic gastric mucosa. **(C)** Moderate NLRP6 staining was observed in gastric cancer tissues. **(D)** Low NLRP6 staining was observed in gastric cancer tissues. **(E)** The overall survival of patients in the NLRP6-high group was significantly longer than that of patients in the NLRP6-moderate and NLRP6-low groups (log-rank test, n=72, χ^2^=8.798, *P*=0.012).

To further investigate the correlation between NLRP6 expression and clinicopathological features as well as the prognostic role of NLRP6 in gastric cancer, 80 paraffin-embedded gastric cancer tissue samples were submitted to immunohistochemical analysis. After measuring NLRP6 expression (Figure 2A-2D), we divided the samples into two groups: high NLRP6 staining (n=20) and low NLRP6 staining (n=60). Analysis of clinicopathological parameters showed that high NLRP6 expression was strongly correlated with local lymph node metastasis (N stage, *P*=0.027), and TNM stage (*P*=0.018). No correlation was observed between NLRP6 expression and patient's age, gender, tumor invasion and distant metastasis (*P*>0.05; Table 1). Kaplan-Meier survival analysis was used to analyze the relation between NLRP6 expression and disease-specific survival. Patients with high NLRP6 expression had a longer 5-year overall survival (OS) than patients with low NLRP6 expression (*P*=0.012, Figure 2E). The median survival month of were 67 and 19 for high and low NLRP6 expression, respectively.

**Table 1:** Correlation between NLRP6 expression and clinicopathological variables of 80 gastric cancer cases

Clinicopathological parameters	n^a^	NLRP6 expression		χ^2^	*P*-Value
		High	Low		
**All**	80	20	60		
**Age(years)**				0.684	0.408
< 60	26	5	21		
≥60	54	15	39		
**Gender**				0.025	0.875
Male	63	16	47		
Female	17	4	13		
**Tumor infiltration**				2.213	0.529
T1	4	0	4		
T2	6	1	5		
T3	52	14	38		
T4	18	3	15		
**Local lymph node metastasis**				4.893	0.027^*^
N0+N1	35	13	22		
N2+N3	45	7	38		
**Distant metastasis**				1.404	0.236
M0	76	20	56		
M1	4	0	4		
**TNM staging**				5.625	0.018^*^
I+II	32	14	28		
III+IV	48	6	42		

^a^Numbers of cases in each group. ^*^ Statistically significant (*P*<0.05).

### NLRP6 inhibits the growth of gastric cancer cells

**Figure 3:**
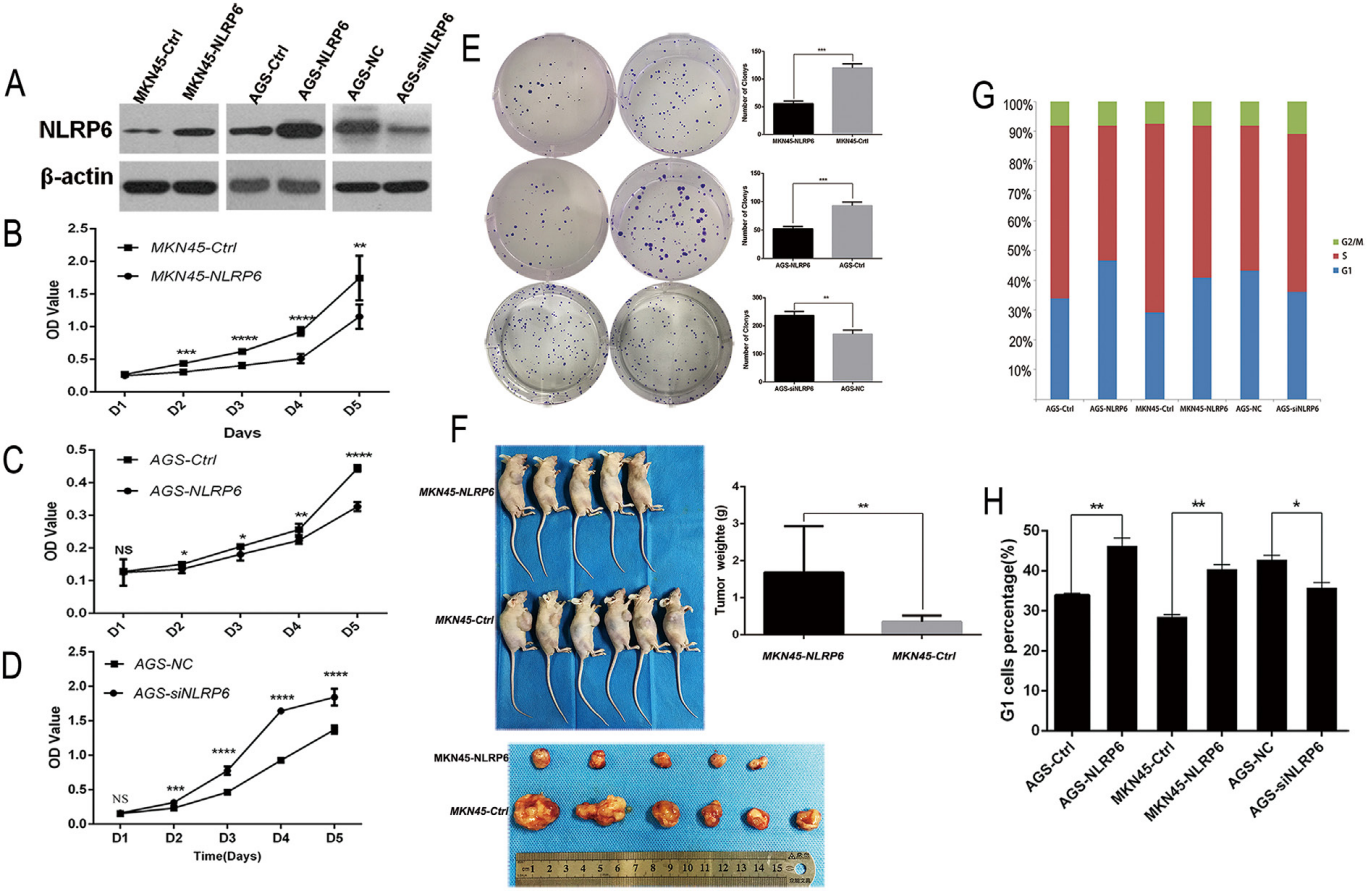
NLRP6 suppresses gastric cancer proliferation *in vitro* and *in vivo* **(A)** NLRP6 protein expression in MKN45 and AGS cells transfected with Ad-NLRP6 or Control and AGS cells transfected with NLRP6 siRNA or Control. **(B-C)** MTT assays were performed to compare the cell growth rates between NLRP6-overexpressing and control cells.**(D)** MTT assays were performed to compare the cell growth rates between NLRP6-silenced and control cells. **(E)** Representative images of the increased colony formation induced by NLRP6 in gastric cancer cell lines. Quantitative analysis of colony formation numbers was performed, and the results are shown in the right panel. **(F)** Tumor weight was decreased in the MKN45 group with stable overexpression of NLRP6 compared to the control group. **(G)** Flow cytometry analysis to assess PI staining of DNA in gastric cancer cells. The percentages of cells in the G1, S, and G2/M phases are shown. **(H)** Gastric cancer cells were stained with PI, and flow cytometry was used to count the number of cells in the G1 phase. The data are shown as the mean ± SD of three independent experiments (^*^P<0.05, ^**^P<0.01, ^***^P<0.001, ^****^P<0.0001).

To determine the effect of NLRP6 on gastric cancer cell proliferation, AGS and MKN45 cells were transfected with either the NLRP6-overexpression or control lentivirus. In addition, AGS cells were transfected with either NLRP6 or negative-control siRNA. The cell proliferation rate was assessed via MTT assays. Western blotting confirmed the overexpression of NLRP6 in the transfected cells (Figure 3A). As shown in Figure 3B-3C, NLRP6 overexpression inhibited the growth of MKN45 and AGS cells, whereas treatment of AGS cells with siRNA accelerated their growth compared with the negative control (*P*<0.05, Figure 3D). Colony formation assays revealed that both MKN45-NLRP6 cells (55.67 ± 2.603) and AGS-NLRP6 cells (52.00 ± 2.646) formed fewer colonies than MKN45-Ctrl cells (120.0 ± 4.359) and AGS-Ctrl cells (93.00 ± 3.786) (*P*<0.001; Figure 3E). AGS-NLRP6 cells transfected with NLRP6-siRNA (236.7 ± 8.413) formed more colonies than the corresponding negative control cells (170.7 ± 8.192) (*P*<0.01; Figure 3E). To examine the role of NLRP6 overexpression *in vivo*, stable MKN45-NLRP6 and MKN45-Ctrl cells were subcutaneously injected into the right axillary space of nude mice. The volumes and weights of the tumors in the MKN45-NLRP6-overexpression group (0.3501 ± 0.07550 g, n=5) were significantly lower than those in the MKN45-Ctrl group (1.688 ± 0.5074 g, n=6, *P*<0.01; Figure 3F).

### NLRP6 suppresses gastric cancer proliferation via P14ARF–Mdm2–P53-dependent cellular senescence

**Figure 4:**
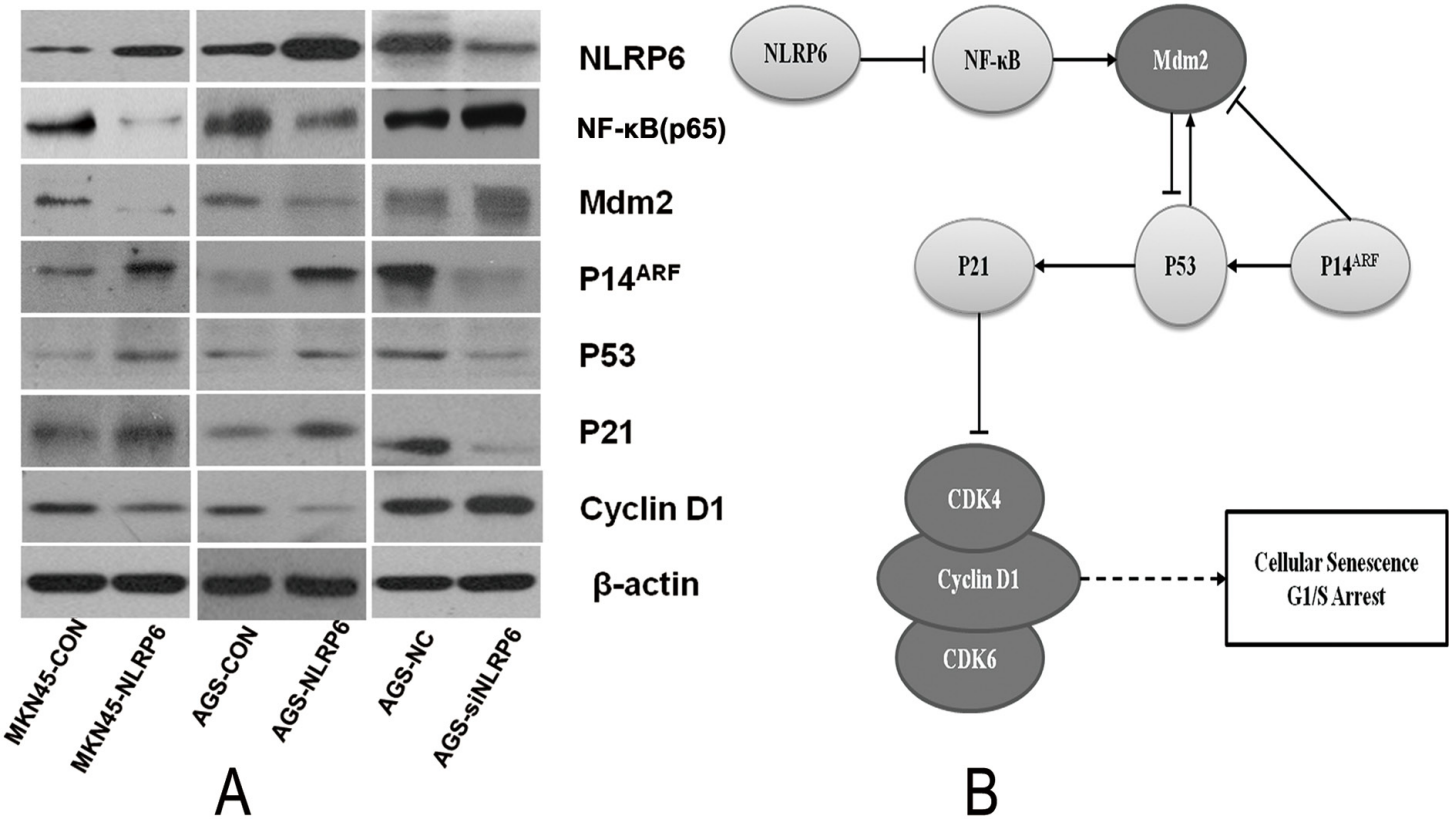
The role of NLRP6 in gastric cancer **(A)** The effects of NLRP6 on the cell cycle determined by Western blot analysis. All protein expression levels were quantitatively analyzed and expressed as ratios to β-actin. The data are representative of three experiments. Protein expression of NLRP6, NF-κB, Mdm2, P14, P53, P21 and Cyclin D1 was evaluated by Western blotting. β-actin was used as an internal control. **(B)** Schematic representation of the role of NLRP6 in gastric cancer.

To explore the mechanism by which NLRP6 suppresses gastric cancer cell growth, we next investigated the effects of NLRP6 overexpression on cell cycle progression in gastric cancer cells using flow cytometry. The results showed that there was a higher percentage of cells in the G1 phase in the NLRP6-overexpression group than in the control group. In contrast, there was a lower percentage of cells in the G1 phase in the NLRP6-knockdown group than in the control group (Figure 3G-3H). Senescent tumor cells may rely on the P14^ARF^-Mdm2-P53 tumor suppressor axis [18]. Therefore, to further assess the mechanism by which NLRP6 suppresses gastric cancer proliferation, we next analyzed the expression of the related proteins by Western blotting. Our data showed that overexpression of NLRP6 was correlated with an increase in P14^ARF^, P53 and P21 expression as well as a decrease in Mdm2 and Cyclin D1 expression (Figure 4A). NLRP6 down-regulation induced the opposite effect. In addition, it has been clearly demonstrated that NLRP6 negatively regulates the NF-κB pathway [13, 19, 20], and inhibition of Mdm2, in turn, inhibits NF-κB activation [21]. Our findings were consistent with these previous observations (Figure 4A), and therefore, we concluded that NLRP6 is a critical activator of the P14^ARF^-Mdm2-P53 tumor suppressor axis in gastric cancer cells.

## DISCUSSION

The NLR family member NLRP6 is highly expressed in intestinal tissue and can aid in diagnosing and determining the prognosis of patients with gastric and intestinal cancers. Prior reports have demonstrated that NLRP6 promotes intestinal homeostasis and protects against the development of colitis and colitis-associated tumorigenesis in mice [8, 10, 13, 14]. However, the function of NLRP6 has not been fully elucidated.

NLRP6 is an important protector against colitis and colitis-associated tumorigenesis [10, 22–24], but few reports have examined the involvement of NLRP6 in gastric cancer. In the present study, we examined NLRP6 expression in gastric cancer tissues and cell lines and found that expression of this protein was dramatically decreased in gastric cancer. In most of the tested clinical gastric cancer samples, the mRNA and protein levels of NLRP6 were markedly diminished. We also assessed NLRP6 expression in gastric cancer tissue samples and found that NLRP6 expression levels were correlated with patient age, local lymph node metastasis, TNM stage, and OS. These findings further illustrated the anti-oncogenic role of NLRP6 in gastric cancer.

To further elucidate the role of NLRP6 in gastric cancer, we examined the association between NLRP6 expression and gastric cancer cell proliferation *in vitro* and *in vivo*. The results showed that overexpression of NLRP6 significantly suppressed cell proliferation and colony formation of MKN45 and AGS cells, whereas down-regulation of NLRP6 promoted cell proliferation and colony formation of AGS cells. Furthermore, overexpression of NLRP6 markedly inhibited tumor growth in nude mice. These results further confirmed the anti-oncogenic role of NLRP6 in gastric cancer and suggested that NLRP6 may protect against tumorigenesis.

Our results also showed that NLRP6 suppressed cell cycle progression by regulating the G1/S transition in gastric cancer cells. It is well known that cell cycle-associated senescence is mediated by the P14^ARF^–Mdm2–P53 tumor suppressor axis [25], with senescence generally characterized by up-regulated P53 and Cyclin-dependent kinase inhibitor 1 (Cdkn1, P21) and down-regulated senescence-associated-Cyclin activity [26]. In addition, it has been clearly demonstrated that NLRP6 negatively regulates the NF-κB pathway [19, 20]. In a previous study, L. monocytogenes infection induced significant p105 phosphorylation (a canonical NF-κB effector), whereas p100 phosphorylation (a non-canonical NF-κB effector) was similar in wild-type and Nlrp6-deficient macrophages. NLRP6 negatively regulates canonical NF-κB activation pathway. The NF-κB subunit RelA (also known as p65) exhibited increased translocation to the nuclei (increased nuclear fraction) in infected Nlrp6-deficient cells, whereas increased levels of the non-canonical effector RelB were not observed. We also found that NF-κB (p65) was significantly regulated by over-expression or under-expression of NLPR6. As previously shown, Mdm2 inhibition also inhibits NF-κB activation, which consequently suppresses the production of proinflammatory cytokines in the heart after myocardial infarction [21]. These effects of Mdm2 inhibition are mediated through modulation of NF-κB activation, demonstrating the regulatory role of NF-κB. Additionally, Mdm2 is a vital negative regulator of several pathways involved in P53 stability and activity [27].

The P14^ARF^ protein (P14^ARF^ in humans; P19^ARF^ in mice) serves as a key sensor of hyperproliferative signals generated by activated oncogenes and engages both P53-dependent and P53-independent pathways to protect cells from malignant transformation [28]. While the tumor suppressor ARF is readily degraded in normal cells, it is stabilized to promote P53 function after the loss of Pten. ARF was shown to augment P53 stability by promoting the degradation of Mdm2, a negative regulator of P53 [18]. Previous studies have also revealed that the tumor-suppressive capacity of Stat3 in senescent tumor cells [29] may rely on the P19^ARF^-Mdm2-P53 tumor-suppressor axis [18]. In addition, loss of Stat3 can promote prostate cancer development by bypassing the regulation of senescence through the P19^ARF^-P53 axis [18]. Accordingly, overexpression of P19^ARF^ leads to P53-dependent cell growth arrest and induces senescence [30]. Therefore, tumor cell senescence is mediated by the P14^ARF^-Mdm2-P53 axis.

P53 is essential for P21 induction, as demonstrated by the nearly undetectable levels of P21 in P53/Pten double-null cells [25]. P53, which functions as a transcription factor, is stabilized and activated upon DNA damage. In turn, P53 induces the transcription of many genes including P21^Waf1/Cip1^, which functions to inhibit Cdk proteins, which are essential for entry into the S phase [31]. The P21^Waf1/Cip1^ protein is the only critical downstream target of P53 that can block the G1 cell cycle machinery in response to DNA damage [31]. Cyclin D proteins, including Cyclin D1, D2 and D3, are the first Cyclin proteins to sense mitogenic signals. In their role as growth-factor sensors, these proteins activate CDK4 and CDK6 during the G1 phase [32]. Hyper-activation of these CDKs can cause uninhibited cell division and tumor development. INK family members bind to CDK4 and CDK6 to block their interaction with Cyclin D. CIP/KIP family members bind to the Cyclin D/CDK complex and suppress its catalytic activity, causing G1/S arrest [33–35]. Induction of P21 by P53 upon DNA damage inhibits Cyclin D/CDK2 and Cyclin D/CDK4 and thereby inhibits G1/S transition. Our present work clearly shows that NLRP6 activity inhibits the expression of NF-κB, Mdm2 and Cyclin D1 and induces of the expression of P14^ARF^, P53 and P21. Taken together, our results demonstrated that NLRP6 targeting of P14^ARF^–Mdm2–P53-dependent cellular senescence suppressed gastric tumorigenesis (Figure 4B).

In conclusion, our work showed that NLRP6 significantly inhibited gastric cancer cell proliferation *in vitro* and *in vivo* by arresting cell transition from the G1 to S phase. To the best of our knowledge, these data are the first to indicate a relationship between NLRP6 expression and gastric cancer cell proliferation. Our study provides new insights into the function of NLRP6 and demonstrates that NLRP6 overexpression is a promising strategy for gastric cancer therapy. Moreover, the level of NLRP6 expression can potentially be used as a prognostic determinant in gastric cancer patients. In addition, exploiting the mechanism by which NLRP6 suppresses gastric tumorigenesis via P14^ARF^–Mdm2–P53-dependent cellular senescence may also prove to be a promising strategy for gastric cancer therapy.

## MATERIALS AND METHODS

### Cell lines and culture conditions

The gastric cancer cell lines MKN45, AGS, SGC7901, MGC803 and GES-1 were obtained from the Department of Cancer Center, The First Affiliated of Xiamen University (Xiamen, China). All cells were cultured in RPMI-1640 medium supplemented with 10% heat-inactivated fetal bovine serum (FBS) and maintained at 37°C in a humidified chamber containing 5% CO_2_.

### Patient information and tissue specimens

Gastric cancer tissues and corresponding normal adjacent tissues were obtained from the Department of Gastrointestinal Surgery at The First Affiliated Hospital of Xiamen University (Xiamen, China) from September 2015 to February 2016. All samples were collected with the patients’ informed consent, and all tissues were pathologically confirmed. This study was approved by the Ethics Committee of The First Affiliated Hospital of Xiamen University. Thirty gastric cancer specimens and the corresponding adjacent noncancerous tissues were frozen and stored in liquid nitrogen until further use.

### Immunohistochemistry

Immunohistochemical (IHC) analysis was used to study NLRP6 protein expression in a tissue array (Shanghai Outdo Biotech Co. Ltd., China) that included 72 human paraffin-embedded gastric cancer samples. The procedure was carried out similarly to previously described methods [36]. A primary antibody against NLRP6 was used for detection (1:600 dilution; Millipore, USA). The degree of immunostaining of the formalin-fixed, paraffin-embedded sections was reviewed and scored independently by 2 observers using a high-power (×200) microscope. The value was based on the staining intensity score (0=negative, 1=weak, 2=moderate, 3=strong) and the proportion of positively stained cells among the total cell population (proportion score: 0 < 10%, 100≤1 < 30%, 30%≤2 < 60%, 60%≤3≤100%). The latter score was acquired based on the difference between normal adjacent tissues and gastric cancer tissues. We defined an overall score of 0-1 as high NLRP6 expression, a score of 2 as moderate NLRP6 expression, and a score of 3-5 as low NLRP6 expression.

### Quantitative real-time PCR (qRT-PCR)

qRT-PCR was performed as previously described [37]. Total RNA was isolated using TRIzol (Invitrogen, USA) according to the manufacturer’s instructions. For qRT–PCR analysis, 500 ng of total RNA was reverse-transcribed to cDNA using PrimeScript™ RT Master Mix (Takara, Japan). qRT–PCR was performed in triplicate with SYBR® Premix Ex Taq™ II (Takara, Japan). Real-time monitoring of PCR amplification was performed using the QuantStudio™ 7 Flex Real-Time PCR System (Life Technologies, USA). GAPDH was used as an internal control for NLRP6. The reverse-transcription primers and qRT-PCR primers used for NLRP6 and GAPDH were purchased from Sangon Biotech (Guangzhou, China). The relative expression of NLRP6 was evaluated using the 2^-∆∆^CT method. The expression levels of NLRP6 in gastric cancer tissue samples were calculated as ^∆∆^CT=*C*_*Tumor*_(*C*_T, NLRP6_ –*C*_T, GAPDH_) – *C*_Normal_ (*C*_T, NLRP6_–*C*_T, GAPDH_). All experiments were performed at least three times. The following primers were used: NLRP6 5'-AAGGAACTGGAGCAACTG-3' and 5'-CGATGAACTGGTAGGTGAC-3'; GAPDH 5'-ACAACTTTGGTATCGTGGAAGG-3' and 5'-GCCATCACGCCACAGTTTC-3'.

### Western blotting

The gastric cancer tumor tissues and tumor-adjacent normal tissues and the gastric cancer cell lines were lysed in RIPA lysis buffer, and the lysates were harvested by centrifugation (12,000 rpm) at 4°C for 30 min. Protein samples of approximately 20 μg each were then resolved by sodium dodecyl sulfate-polyacrylamide gel electrophoresis on 12% gels and transferred to a PVDF membranes. After blocking the non-specific binding sites for 60 min with 5% non-fat milk, the membranes were incubated overnight at 4°C with anti-human antibodies to NLRP6 (1:1,000; Millipore, USA), NF-kB, Caspase-1, P53, Mdm2, P14, P21, Cyclin D1 (1:1,000; Cell Signaling Technology, USA), or β-actin (at 1:50,000; Sigma, USA). The membranes were then washed three times with Tris-buffered saline with Tween-20 (TBST) for 10 min each time. After washing, the membranes were probed with a horseradish peroxidase-conjugated secondary antibody. Signals on the membranes were detected using an ECL prime kit (Millipore, USA), and images were obtained with a LumiCube (Liponics, Japan). All experiments were performed at least three times.

### Establishment of cells stably overexpressing NLRP6

Stable NLRP6-overexpressing gastric cancer cell lines were generated using previously described protocols [38]. The NLRP6-containing flank region was amplified from human genomic DNA and inserted into pUbi-MCS-3FLAG-SV40-EGFP-IRES-puromycin (GeneChem, China). A lentivirus-mediated packaging system containing four plasmids, pUbi-NLRP6 or control plasmid, pMDL, REV, and VSVG, was used to establish stable NLRP6-expressing gastric cancer cell lines. The gastric cancer cells were seeded at a density of 1.0 × 10^4^ cells/well in a 6-well plate and inoculated with a recombinant lentivirus at a suitable multiplicity of infection (MOI) in the presence of 50 μg/mL polybrene (MKN45-NLRP6 and AGS-NLRP6). Gastric cancer cells transfected with a control plasmid were established as a control (MKN45-Ctrl and AGS-Ctrl). Cells transduced with the above lentiviruses were maintained using 2 μg/mL puromycin for at least 2 weeks before each experiment.

### Loss-of-function analysis using small interfering RNA (siRNA)

For loss-of-function analysis, the transfection of NLRP6 siRNA (oligo sense: 5'-UCACCAAGCGCUUCACCAATT-3', antisense: 5'-UUGGUGAAGCGCUUGGUGATT-3') and negative control siRNA (oligo sense: 5'-UUCUCCGAACGUGUCACGUTT-3', antisense: 5'-ACGUGACACGUUCGGAGAATT-3') was performed using Lipofectamine 2000 (Invitrogen, USA) following the manufacturer’s protocol. Cells were maintained for 48 h after transfection. Then, NLRP6 expression was detected by Western blotting. The cells subjected to knockdown were used in subsequent experiments.

### Proliferation assay

An MTT assay was used to evaluate cell proliferation. Cells were seeded in 96-well plates at a density of 2×10^3^ cells/well. Samples were collected after the 1^st^, 2^nd^, 3^rd^, 4^th^ and 5^th^ day of seeding. MTT was added to each well, and the absorbance at 490 nm was measured 4 h later. All experiments were performed at least three times.

### Colony formation assay

To assess colony formation, cells were seeded in a 6-well plate at a density of 2×10^2^ cells/well. After 10-14 days of cultivation, surviving colonies (>50 cells per colony) were fixed with 4% paraformaldehyde for 20 min, stained with 0.1% crystal violet for 15 min, and photographed using a digital camera. Colony-forming efficiency (CFE %) was defined as the ratio of the number of colonies formed in culture to the number of cells inoculated. All experiments were performed at least three times.

### Cell cycle analysis

Flow cytometry analysis was performed as previously described [39]. Cells were seeded overnight on 60-mm plates in complete medium with 10% FBS. After washing with ice-cold PBS three times, the cells were harvested by trypsinization and fixed overnight in 1 ml of 70% alcohol at 4°C. After fixation, the cells were again washed three times with ice-cold PBS and then filtered through 50-mm nylon mesh. Cell pellets were resuspended in Cell Cycle Kit reagent, incubated for 30 min, and protected from light until further analysis using a flow cytometer (EPICS XL; Coulter, Miami, FL). The cell cycle was analyzed using Multicycle-DNA Cell Cycle Analyzed Software. The proliferation index (PI) was calculated as PI=(S+G2)/(S+G2+G1). All experiments were performed at least three times.

### Animal experiments

All experiments using animals were performed in accordance with a protocol approved by the Animal Care and Use Committee of Xiamen University. To assess tumorigenicity *in vivo*, a cell suspension of 5×10^6^ of either stable cells overexpressing NLRP6 or control cells was subcutaneously injected into nude mice (n=5/6). The mice were euthanized 30 days after the injection, and tumor weight was measured.

### Statistical analysis

SPSS 21.0 software was used for statistical analysis. All images were generated using GraphPad Prism 6. Results are reported as the mean ± SD of three or more independent experiments. Comparisons were performed using a two-tailed paired Student’s t test. P<0.05 was considered statistically significant.
