# Correction: NLRP6 targeting suppresses gastric tumorigenesis via P14^ARF^–Mdm2–P53-dependent cellular senescence

**DOI:** 10.18632/oncotarget.26283

**Published:** 2018-10-26

**Authors:** Haibin Wang, Guoxing Xu, Zhengjie Huang, Weizheng Li, Huali Cai, Yunda Zhang, Disheng Xiong, Gang Liu, Shengjie Wang, Zengfu Xue, Qi Luo

**Affiliations:** ^1^ Department of Gastrointestinal Surgery, Xiamen Cancer Hospital, The First Affiliated Hospital of Xiamen University, Xiamen 361003, Fujian, China; ^2^ Department of Endoscopy Center, The First Affiliated Hospital of Xiamen University, Xiamen 361003, Fujian, China; ^3^ Department of Gastrointestinal Surgery, First Clinical Medical College of Fujian Medical University, Fuzhou 350004, China; ^4^ Department of Cancer Prevention, Diagnosis and Treatment, Xiamen Cancer Hospital, The First Affiliated Hospital of Xiamen University, Xiamen 361003, Fujian, China

**This article has been corrected:** The correct Title information is given below:

**NLRP6 targeting suppresses gastric tumorigenesis via P14^ARF^–Mdm2–P53-dependent cellular senescence**

Original article: Oncotarget. 2017; 8:111597-111607. https://doi.org/10.18632/oncotarget.22876

